# Association Between Cholesterol‐HDL‐Glucose (CHG) Index and New‐Onset Hypertension Across Different Sex and Glycemic Status: A Longitudinal Study With 9‐Year Follow‐Up

**DOI:** 10.1002/edm2.70365

**Published:** 2026-11-03

**Authors:** Huabin He, Jianhai Chen

**Affiliations:** ^1^ JiuJiang City Key Laboratory of Cell Therapy JiuJiang NO. 1 People's Hospital Jiujiang China; ^2^ Department of Cardiovascular Medicine Jiujiang NO. 1 People's Hospital Jiujiang China

**Keywords:** CHARLS, CHG index, hypertension, IDI, NRI, TyG index

## Abstract

**Background:**

Hypertension is a major global public health issue, and the continuous search for new biomarkers to optimize risk stratification is crucial. This study aims to investigate the relationship between a novel metabolic disorder diagnostic tool—cholesterol, high‐density lipoprotein, and glucose (CHG) index—and the incidence of hypertension in populations with different sexes and glycemic statuses.

**Methods:**

This study utilized 9 years of follow‐up data from the China Health and Retirement Longitudinal Study. Logistic regression models were employed to examine the association between the CHG index and new‐onset hypertension across different sex and glycemic statuses. Additionally, the net reclassification improvement and integrated discrimination improvement indices were calculated to estimate the additional predictive value beyond the basic model.

**Results:**

A total of 6555 participants were included in the study. A significant association between CHG index and new‐onset hypertension was observed in the total, male, female, prediabetic, and diabetic populations, with adjusted OR (95% CI) of 1.16 (1.10–1.24), 1.23 (1.12–1.36), 1.12 (1.02–1.22), 1.14 (1.01–1.29), and 1.19 (1.03–1.36) respectively. Restricted Cubic Spline (RCS) analysis revealed a positive linear relationship between the CHG index and new‐onset hypertension. The addition of either the CHG index or the Triglyceride‐Glucose (TyG) index significantly improved the predictive performance of the basic model for hypertension.

**Conclusions:**

An elevated CHG index is significantly associated with new‐onset hypertension.

## Graphical abstract

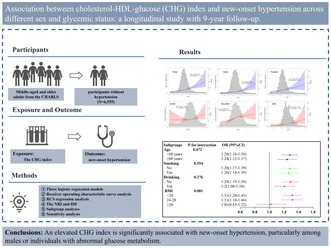

## 1 Introduction

Hypertension has become a major global public health problem. According to data from the World Health Organization, there are approximately 1.4 billion people worldwide living with hypertension, with the majority located in developing countries [1]. ‘China Cardiovascular Health and Disease Report 2023’ points out that the number of hypertension patients in China has climbed to 245 million [2]. As a core risk factor for cardiovascular disease (CVD), hypertension significantly increases the risk of morbidity and mortality from coronary heart disease, stroke, kidney disease, and other vascular diseases [3]. Hypertension is not only a major modifiable risk factor for CVD and all‐cause mortality but also a key area for public health intervention [4]. Identifying at‐risk populations and implementing early interventions are essential to fundamentally improve hypertension status. Therefore, it is particularly important to continue to explore new biomarkers to optimize risk stratification for hypertension.

Epidemiological studies have shown that individuals with Type 2 diabetes often have concomitant hypertension, and those with insulin resistance and other metabolic disturbances tend to have a higher prevalence of hypertension [5]. Based on this association, several biomarkers have been proposed to optimize risk stratification for new‐onset hypertension [6, 7, 8, 9, 10]. For instance, the widely recognized Triglyceride‐Glucose (TyG) index and its derived indices have been demonstrated to be important tools for predicting hypertension risk and prognosis [10]. Notably, a recent study by Mansoori and colleagues introduced a novel biomarker—the Cholesterol, High‐Density Lipoprotein, and Glucose (CHG) index [11]. Research indicated that the CHG index has superior diagnostic efficacy over the TyG index in diagnosing Type 2 diabetes [11]. Additionally, one study has found that elevated CHG index is significantly associated with CVD risk and exhibits predictive power comparable to the TyG index in assessing CVD risk [12]. However, no studies have yet investigated the relationship between the CHG index and new‐onset hypertension, particularly in individuals of different sex and glycemic statuses. Moreover, it remains unverified whether incorporating the CHG index into basic predictive models could enhance their predictive capability.

To fill these critical research gaps, this study utilizes 9 years of follow‐up data from the China Health and Retirement Longitudinal Study (CHARLS) to investigate the relationship between the CHG index and new‐onset hypertension across different sex and glycemic status groups. Additionally, we assess whether incorporating the CHG index into a basic predictive model can enhance its predictive capacity and include comparative analyses with the TyG index.

## 2 Methods

### 2.1 Study Population

Our study data was derived from CHARLS. CHARLS is a national survey targeting Chinese residents aged 45 and above [13]. The study employs a multilevel stratified sampling method and utilizes standardized questionnaires to collect data from middle‐aged and elderly individuals across 28 provinces, 150 districts, and 450 villages in China. Wave 1 of the survey began in 2011, with follow‐up surveys conducted every 2 to 3 years thereafter. This study adhered to the principles outlined in the Helsinki Declaration and was approved by the Biomedical Ethics Committee of Peking University (IRB00001052‐11015). All participants provided written informed consent. For more detailed information about CHARLS, please visit: http://charls.pku.edu.cn/.

In this study, we used 2011 Wave 1 data as baseline and 2020 Wave 5 data as follow‐up. There were 17,705 participants in Wave 1. The exclusion criteria were as follows: (1) participants aged under 45 years; (2) participants with missing data for assessing glycemic status; (3) participants with missing data for calculating TyG index and CHG index; (4) participants with missing hypertension diagnosis data or those diagnosed with hypertension at baseline (Hypertension was defined as systolic blood pressure ≥ 140 mm Hg, diastolic blood pressure ≥ 90 mm Hg, or a self‐reported history of hypertension.); (5) participants with missing hypertension diagnosis data at wave 5. A total of 6555 participants were included in the final analysis. The detailed participant inclusion and exclusion process was shown in Figure S1.

### 2.2 CHG Index and TyG Index Assessment

The CHG index was calculated using the formula: CHG index = Ln[fasting plasma glucose (mg/dL) × total cholesterol (mg/dl)/(2 × high‐density lipoprotein cholesterol (mg/dL))] [11]. The TyG index is calculated as: TyG index = Ln [fasting triglycerides (mg/dL) × fasting blood glucose (mg/dL)/2] [14].

### 2.3 Assessment of New‐Onset Hypertension

Since blood pressure measurement data were not included in the Wave 5 survey, this study assessed hypertension status using a self‐reported questionnaire based on prior research [8]. We excluded participants who reported a physician‐diagnosed history of hypertension at the 2011 baseline. Incident hypertension was defined as the first self‐reported, physician‐confirmed diagnosis during any of the follow‐up surveys conducted in 2013, 2015, 2018, or 2020. To enhance case ascertainment and minimize recall bias, all self‐reported events across the follow‐up waves were aggregated and further verified against the retrospective disease history collected in the Wave 5 (2020) questionnaire. All participants were asked the standardized question: ‘Have you ever been diagnosed with hypertension by a doctor?’ Those who answered ‘Yes’ were classified as having hypertension.

### 2.4 Covariate Evaluation and Definitions

This study collected various demographic data at baseline, including age, sex, residence, drinking, smoking, marital status, and education level. Additionally, participants' physical measurement data were recorded, such as weight, height, and body mass index (BMI). Self‐reported medical histories of chronic conditions were also included, covering diabetes mellitus (DM), liver disease, chronic lung diseases, asthma, digestive disorders, dyslipidemia, kidney disease, arthritis, mental health disorders, memory‐related diseases, and cancer. Laboratory test data were collected, including fasting plasma glucose (FPG), triglycerides (TG), total cholesterol (TC), glycated haemoglobin (HbA1c), uric acid (UA), and C‐reactive protein (CRP) levels. Pre‐diabetes mellitus (pre‐DM) was defined as an HbA1c level between 5.7% and 6.4%, or a FPG level between 5.6 and 6.9 mmol/L [15]. Normal glucose regulation (NGR) was defined as an HbA1c < 5.7% and FPG < 5.6 mmol/L [15]. DM was defined as self‐reported diabetes, or an HbA1c ≥ 6.5% or FPG ≥ 7.0 mmol/L [15].

### 2.5 Statistical Analysis

Participants were divided into four groups according to the quartiles of the CHG index. Baseline characteristics of participants were summarized using analysis of variance, Kruskal‐Wallis test, and chi‐square test, as appropriate.

We fitted three logistic regression models to assess the association between the CHG index and new‐onset hypertension across different sex and glycemic status groups. In the models, the CHG index was analysed both as a categorical variable defined by quartiles and as a continuous variable. Specifically, Model 1 was unadjusted; Model 2 was adjusted for sex, age, drinking, smoking, residence, BMI, and education level; and Model 3 further adjusted for potential confounders, including DM, liver disease, chronic lung diseases, asthma, digestive disorders, dyslipidemia, kidney disease, arthritis, mental health disorders, memory‐related diseases, cancer, systolic blood pressure (SBP), and diastolic blood pressure (DBP). This study employed the variance inflation factor (VIF) to test for multicollinearity among all included variables, so as to eliminate the interference of collinearity on regression results and ensure the stability and reliability of the model. The results indicated no significant multicollinearity for all variables, as shown in Table S1. Missing values for covariates were imputed using the mice package in R. The missing rate of each covariate prior to imputation is shown in Table 1. Predictive mean matching (PMM) was used for continuous variables, and logistic‐regression‐based imputation was performed for binary variables. Five imputed datasets were generated, and regression estimates from each dataset were pooled using Rubin's rules. Additionally, the relationship between the TyG index and new‐onset hypertension was also examined in this study population.

**TABLE 1:** Comparison of baseline characteristics stratified by CHG index quartiles.

Characteristics	Overall	CHG quartiles				*p*
		Q1	Q2	Q3	Q4	
Participants, N	6555	1636	1641	1632	1646	
Age, years	57.30 ± 8.46	57.12 ± 8.74	57.24 ± 8.50	57.37 ± 8.48	57.48 ± 8.12	0.192
Male, *n* (%)	3016 (46.0)	788 (48.2)	721 (43.9)	760 (46.6)	747 (45.4)	0.095
Smoking^a^, *n* (%)	2517 (38.4)	664 (40.6)	605 (36.9)	620 (38.0)	628 (38.2)	0.163
Drinking^a^, *n* (%)	2002 (30.6)	589 (36.0)	482 (29.4)	483 (29.6)	448 (27.2)	< 0.001
Marital status (*n*, %)						
Partnered	5953 (90.8)	1479 (90.4)	1479 (90.1)	1479 (90.6)	1516 (92.1)	0.203
Single	602 (9.2)	157 (9.6)	162 (9.9)	153 (9.4)	130 (7.9)	
Rural (vs urban)^a^, *n* (%)	5569 (85.0)	1459 (89.2)	1412 (86.0)	1372 (84.1)	1326 (80.6)	< 0.001
Educational level^a^, *n* (%)						
Illiterate	2971 (45.3)	772 (47.2)	755 (46.0)	715 (43.8)	729 (44.3)	0.354
Primary school	1445 (22.1)	366 (22.4)	365 (22.2)	362 (22.2)	352 (21.5)	
Middle school	1432 (21.9)	343 (21.0)	354 (21.6)	368 (22.6)	367 (22.3)	
High school or above	702 (10.7)	154 (9.4)	167 (10.2)	186 (11.4)	195 (11.9)	
BMI (kg/m^2^)	23.0 (21.0, 25.0)	21.0 (20.0, 23.0)	23.0 (22.0, 24.0)	23.0 (21.0, 26.0)	24.0 (22.0, 27.0)	< 0.001
Glycemic status, *n* (%)						
NGR	2757 (42.1)	1146 (70.1)	815 (49.6)	586 (35.9)	210 (12.8)	< 0.001
Pre‐diabetes	2893 (44.1)	447 (27.3)	756 (46.1)	857 (52.5)	833 (50.6)	
Diabetes	905 (13.8)	43 (2.6)	70 (4.3)	189 (11.6)	603 (36.6)	
Arthritis, *n* (%)	2216 (33.9)	561 (34.3)	565 (34.4)	552 (33.8)	538 (32.7)	0.713
Stroke^a^, *n* (%)	64 (1.0)	14 (0.9)	17 (1.0)	13 (0.8)	20 (1.2)	0.607
Heart disease^a^, *n* (%)	488 (7.5)	102 (6.2)	117 (7.1)	127 (7.8)	142 (8.6)	0.063
Dyslipidemia^a^, *n* (%)	354 (5.5)	44 (2.7)	66 (4.0)	90 (5.5)	154 (9.4)	< 0.001
Cancer^a^, *n* (%)	57 (0.9)	10 (0.6)	17 (1.0)	17 (1.0)	13 (0.8)	0.481
Liver disease^a^, *n* (%)	228 (3.5)	55 (3.4)	74 (4.5)	47 (2.9)	52 (3.2)	0.058
Chronic Lung disease^a^, *n* (%)	553 (8.5)	167 (10.2)	132 (8.0)	130 (8.0)	124 (7.5)	0.026
Digestive disease^a^, *n* (%)	1569 (24.0)	421 (25.7)	421 (25.7)	394 (24.1)	333 (20.2)	< 0.001
Kidney disease^a^, *n* (%)	388 (5.9)	122 (7.5)	94 (5.7)	82 (5.0)	90 (5.5)	0.017
Asthma^a^, *n* (%)	196 (3.0)	58 (3.6)	46 (2.8)	50 (3.1)	42 (2.6)	0.378
Memory‐related disease^a^, *n* (%)	47 (0.7)	16 (1.0)	11 (0.7)	8 (0.5)	12 (0.7)	0.421
Psychiatric disorders^a^, *n* (%)	85 (1.3)	25 (1.5)	23 (1.4)	16 (1.0)	21 (1.3)	0.549
CRP, mg/dL	0.90 (0.50, 1.85)	0.71 (0.41, 1.53)	0.77 (0.46, 1.59)	0.92 (0.54, 1.82)	1.25 (0.68, 2.42)	< 0.001
UA, mg/dL	4.32 ± 1.18	4.14 ± 1.11	4.20 ± 1.14	4.33 ± 1.13	4.62 ± 1.27	< 0.001
TG, mg/dL	100.89 (72.57, 147.79)	70.80 (55.80, 92.00)	88.50 (69.90, 115.00)	114.00 (85.80, 150.00)	173.00 (119.00, 249.00)	< 0.001
FPG, mg/dL	93.96 (101.70, 111.42)	93.80 (87.10, 100.00)	99.40 (92.90, 106.00)	102.00 (96.10, 111.00)	115.00 (104.00, 138.00)	< 0.001
TC, mg/dL	192.29 ± 37.35	175.18 ± 31.58	186.44 ± 31.95	196.15 ± 34.28	211.31 ± 41.04	< 0.001
HDL‐C, mg/dL	51.99 ± 15.24	65.97 ± 15.00	55.07 ± 11.07	47.74 ± 10.03	39.26 ± 9.82	< 0.001

^a^ Missing data: 5 for education level, 2 for drinking, 2 for smoking, 3 for residence, 919 for BMI, 11 for arthritis, 13 for stroke, 20 for heart disease, 71 for dyslipidemia, 14 for cancer, 29 for liver disease, 15 for chronic Lung disease, 10 for digestive disease, 25 for kidney disease, 23 for asthma, 18 for memory‐related disease, 24 for psychiatric disorders.

To more comprehensively evaluate the dose–response relationship between the CHG index and incident hypertension, we fitted restricted cubic spline (RCS) regression models with three knots. To compare the predictive performance of the CHG index and the TyG index for incident hypertension, we performed receiver operating characteristic (ROC) curve analysis, used the C‐statistic to quantify model discrimination, and applied the Hosmer‐Lemeshow goodness‐of‐fit test and calibration curves to assess model calibration (Figure S2). To further assess whether the continuous CHG index provided incremental predictive value beyond the basic model—which included sex, age, drinking status, smoking status, residence, BMI, educational level, liver disease, chronic lung disease, asthma, digestive disorders, dyslipidaemia, kidney disease, arthritis, mental health disorders, memory‐related diseases, and cancer—we calculated the Net Reclassification Improvement (NRI) and Integrated Discrimination Improvement (IDI) indices. The NRI quantifies the net proportion of participants with correctly reclassified predicted risk after adding the new biomarker; the IDI reflects the overall improvement in model discrimination by comparing the differences in predicted risk between cases and non‐cases [16].

To explore the applicability of the CHG index in different populations, we also performed subgroup analyses. These subgroups included age (< 60 years/≥ 60 years), BMI (<  24, 24–28, and ≥ 28 kg/m^2^), smoking (yes/no), and drinking (yes/no). To assess the robustness of the results, we performed two sensitivity analyses on top of Model 3, adjusting for laboratory indicators (UA and CRP) and for medication use (lipid‐lowering, glucose‐lowering, and antihypertensive drugs), respectively, and re‐examined the association between the CHG index and incident hypertension.

All statistical analyses were performed using R software version 4.2.0. A two‐sided *p*‐value of less than 0.05 was considered statistically significant.

## 3 Results

### 3.1 Population Characteristics

A total of 6555 participants were included in the study (3016 males and 3539 females). The mean age of participants was 57.30 ± 8.46 years. Table 1 shows a comparison of baseline characteristics stratified by CHG index quartiles. Individuals with higher CHG quartiles were more likely to be nonsmokers, urban residents, and tended to have dyslipidemia, chronic lung disease, kidney disease, and digestive disorders than those with lower CHG quartiles. In addition, higher CHG quartiles were associated with increased CRP, TG, TC, UA, FPG, and BMI.

To assess the potential for selection bias, we further compared the sociodemographic characteristics of CHARLS participants aged ≥ 45 years who were included in the present study with those who were excluded. The results are presented in Table S2.

### 3.2 Association Between the CHG Index and New‐Onset Hypertension

During the 9‐year follow‐up period from 2011 to 2020, a total of 2055 participants developed new‐onset hypertension. Table 2 shows the association between the CHG index and new‐onset hypertension events. In the overall population, as well as in both male and female subgroups, an increase in the CHG index was significantly associated with new‐onset hypertension, regardless of whether confounding factors were adjusted for. After adjusting for various confounders, compared to Q1 of the CHG index, the adjusted odds ratios (ORs) and 95% confidence intervals (CIs) for Q2–Q4 were as follows: for the total population, 1.28 (1.06–1.54), 1.31 (1.09–1.58), and 1.53 (1.27–1.84); for males, 1.11 (0.84–1.46), 1.23 (0.93–1.61), and 1.73 (1.31–2.29); for females, 1.46 (1.13–1.88), 1.42 (1.10–1.84), and 1.43 (1.10–1.85). Moreover, when the CHG index was treated as a continuous variable, this association remained significant. Similarly, we found a significant association between elevated TyG index and new‐onset hypertension in this study population (Table S3).

**TABLE 2:** Association between the CHG index and new‐onset hypertension events.

Categories	Mode 1		Mode 2		Mode 3	
	OR (95% CI)	*p*	OR (95% CI)	*p*	OR (95% CI)	*p*
Total						
Continues (Per SD increase)	1.33 (1.26–1.40)	< 0.001	1.28 (1.21–1.35)	< 0.001	1.16 (1.10–1.24)	< 0.001
Quartiles						
Q1	Ref		Ref		Ref	
Q2	1.37 (1.17–1.60)	< 0.001	1.33 (1.13–1.56)	< 0.001	1.28 (1.06–1.54)	< 0.001
Q3	1.73 (1.49–2.02)	< 0.001	1.62 (1.38–1.89)	< 0.001	1.31 (1.09–1.58)	< 0.001
Q4	2.19 (1.89–2.56)	< 0.001	1.96 (1.67–2.30)	< 0.001	1.53 (1.27–1.84)	< 0.001
*P* for trend		< 0.001		< 0.001		< 0.001
Male						
Continues (Per SD increase)	1.33 (1.23–1.44)	< 0.001	1.32 (1.22–1.43)	< 0.001	1.23 (1.12–1.36)	< 0.001
Quartiles						
Q1	Ref		Ref		Ref	
Q2	1.09 (0.86–1.37)	0.485	1.09 (0.86–1.39)	0.460	1.11 (0.84–1.46)	0.477
Q3	1.59 (1.27–1.98)	< 0.001	1.56 (1.25–1.96)	< 0.001	1.23 (0.93–1.61)	0.143
Q4	2.16 (1.73–2.69)	< 0.001	2.08 (1.65–2.63)	< 0.001	1.73 (1.31–2.29)	< 0.001
*P* for trend		< 0.001		< 0.001		< 0.001
Female						
Continues (Per SD increase)	1.33 (1.24–1.43)	< 0.001	1.23 (1.14–1.33)	0.001	1.12 (1.02–1.22)	0.013
Quartiles						
Q1	Ref		Ref		Ref	
Q2	1.65 (1.33–2.05)	< 0.001	1.55 (1.24–1.93)	< 0.001	1.46 (1.13–1.88)	0.003
Q3	1.88 (1.52–2.34)	< 0.001	1.64 (1.31–2.05)	< 0.001	1.42 (1.10–1.84)	0.007
Q4	2.25 (1.82–2.78)	< 0.001	1.83 (1.47–2.29)	< 0.001	1.43 (1.10–1.85)	0.007
*P* for trend		< 0.001		< 0.001		< 0.001

*Note:* Model 1 was unadjusted; Model 2 was adjusted for sex, age, drinking, smoking, residence, BMI, and education level; Model 3 further adjusted for potential confounders, including DM, liver disease, chronic lung diseases, asthma, digestive disorders, dyslipidemia, kidney disease, arthritis, mental health disorders, memory‐related diseases, cancer, systolic blood pressure, and diastolic blood pressure.

Table 3 presents the association between the CHG index and new‐onset hypertension events in different glycemic status groups. After fully adjusting for covariates (Model 3), compared to Q1 of the CHG index, the adjusted ORs and 95% CIs for Q2–Q4 were as follows: for the NGR group, 1.23 (0.96–1.57), 1.1 (0.83–1.45), and 1.16 (0.78–1.70); for the pre‐DM group, 1.37 (1.01–1.89), 1.43 (1.05–1.96), and 1.53 (1.11–2.11); for the DM group, 0.99 (0.69–2.91), 1.15 (0.89–2.84), and 1.59 (1.23–2.57). In the NGR group, no significant association was observed between the CHG index and incident hypertension. In the DM group, only Q4 of the CHG index was significantly associated with hypertension. Notably, when the CHG index was analysed as a continuous variable, significant associations with incident hypertension were consistently observed among participants with prediabetes and diabetes, whereas the evidence for an association in participants with normal glucose metabolism was limited.

**TABLE 3:** Association between the CHG index and new‐onset hypertension events in different glycemic status groups.

Categories	Mode 1		Mode 2		Mode 3	
	OR (95% CI)	*p*	OR (95% CI)	*p*	OR (95% CI)	*p*
NGR						
Continues (Per SD increase)	1.22 (1.08–1.37)	0.001	1.14 (1.01–1.29)	0.040	1.01 (0.87–1.16)	0.983
Quartiles						
Q1	Ref		Ref		Ref	
Q2	0.89 (0.69–1.15)	0.373	0.87 (0.67–1.12)	0.281	1.23 (0.96–1.57)	0.102
Q3	1.41 (1.11–1.79)	0.005	1.33 (1.04–1.71)	0.022	1.1 (0.83–1.45)	0.522
Q4	1.34 (1.05–1.70)	0.019	1.18 (0.92–1.52)	0.194	1.16 (0.78–1.70)	0.470
*P* for trend		0.034		0.219		0.406
Pre‐DM						
Continues (Per SD increase)	1.31 (1.18–1.44)	< 0.001	1.27 (1.15–1.41)	< 0.001	1.14 (1.01–1.29)	0.038
Quartiles						
Q1	Ref		Ref		Ref	
Q2	1.22 (0.97–1.54)	0.083	1.22 (0.96–1.54)	0.101	1.37 (1.01–1.89)	0.048
Q3	1.61 (1.29–2.01)	< 0.001	1.58 (1.25–1.99)	< 0.001	1.43 (1.05–1.96)	0.024
Q4	1.75 (1.40–2.19)	< 0.001	1.67 (1.32–2.12)	< 0.001	1.53 (1.11–2.11)	0.009
*P* for trend		< 0.001		< 0.001		0.016
DM						
Continues (Per SD increase)	1.26 (1.12–1.41)	< 0.001	1.23 (1.09–1.39)	0.001	1.19 (1.03–1.36)	0.016
Quartiles						
Q1	Ref		Ref		Ref	
Q2	1.29 (0.87–1.89)	0.202	1.21 (0.81–1.80)	0.347	0.99 (0.69–2.91)	0.655
Q3	1.22 (0.83–1.80)	0.307	1.1 (0.73–1.65)	0.652	1.15 (0.89–2.84)	0.756
Q4	2.17 (1.48–3.18)	< 0.001	2.08 (1.39–3.13)	< 0.001	1.59 (1.23–2.57)	< 0.001
*P* for trend		0.251		0.297		0.177

*Note:* Model 1 was unadjusted; Model 2 was adjusted for sex, age, drinking, smoking, residence, BMI, and education level; Model 3 further adjusted for potential confounders, including DM, liver disease, chronic lung diseases, asthma, digestive disorders, dyslipidemia, kidney disease, arthritis, mental health disorders, memory‐related diseases, cancer, systolic blood pressure, and diastolic blood pressure.

Further analysis using the RCS model revealed a positive linear relationship between the CHG index and new‐onset hypertension in the overall population, as well as in males, females, individuals with pre‐DM, and those with DM (Figure 1). However, no significant dose–response relationship was observed between the CHG index and developing hypertension in the NGR (Figure 1).

**FIGURE 1:**
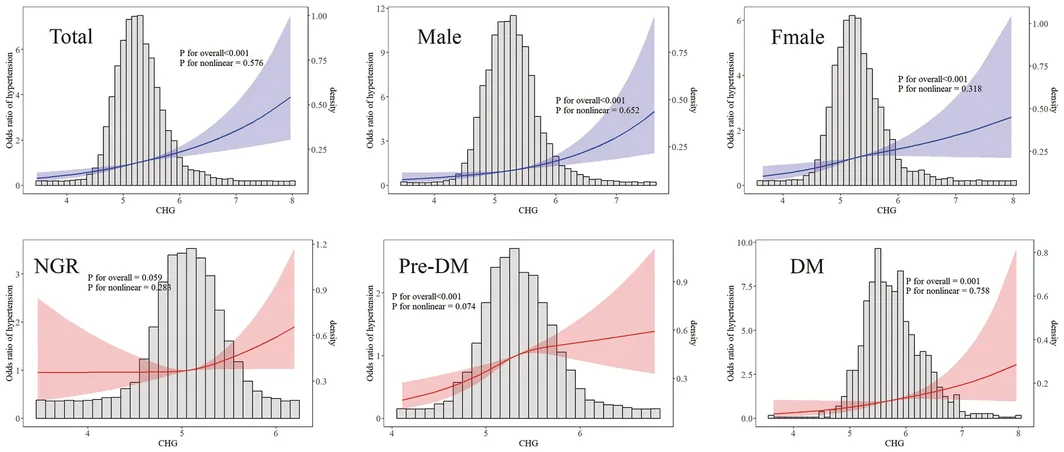
Restricted cubic spline modelling analyzes the relationship between the CHG index and new‐onset hypertension. Adjusted for potential confounders, including sex, age, drinking, smoking, residence, BMI, education level, DM, liver disease, chronic lung diseases, asthma, digestive disorders, dyslipidemia, kidney disease, arthritis, mental health disorders, memory‐related diseases, and cancer.

### 3.3 Incremental Predictive Performance of the CHG Index in New‐Onset Hypertension

Based on Model 3, we constructed a basic prediction model including sex, age, drinking status, smoking status, residence, BMI, educational level, liver disease, chronic lung disease, asthma, digestive disorders, dyslipidemia, kidney disease, arthritis, mental health disorders, memory‐related diseases, and cancer. As shown in Figure 2, adding either the CHG index or the TyG index significantly improved the discrimination of the basic model for incident hypertension (CHG model: C‐statistic = 0.641 vs. 0.624, *p* < 0.001; TyG model: C‐statistic = 0.638 vs. 0.624, *p* < 0.001). Further evaluation using the Net Reclassification Improvement (NRI) and Integrated Discrimination Improvement (IDI) demonstrated that both the CHG index (NRI: 0.197, 95% CI: 0.145–0.249; IDI: 0.011, 95% CI: 0.009–0.014) and the TyG index (NRI: 0.162, 95% CI: 0.110–0.214; IDI: 0.010, 95% CI: 0.007–0.013) significantly improved risk reclassification and model discrimination compared with the basic model (all *p* < 0.001; Table 4). In addition, the results suggest that the CHG index has predictive power comparable to that of the TyG index in predicting new cases of hypertension.

**FIGURE 2:**
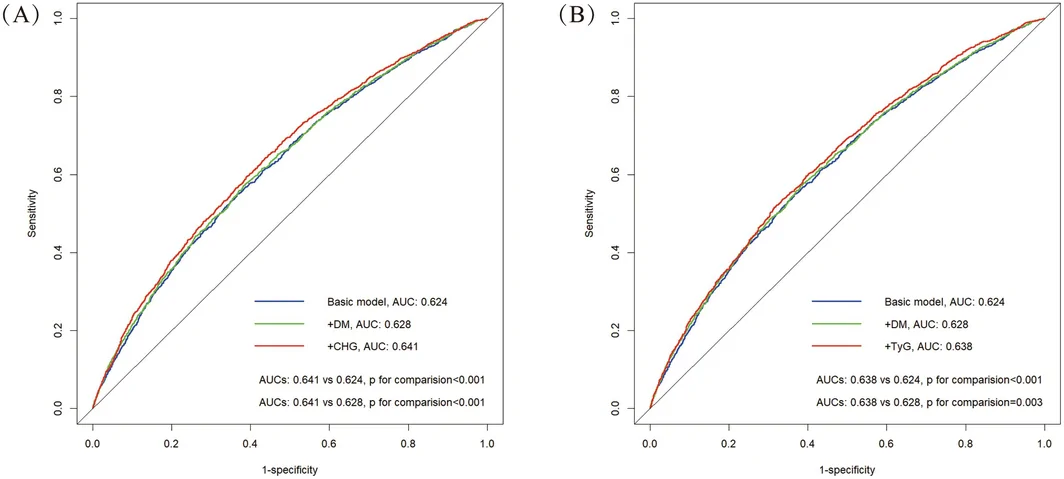
Receiver‐operated characteristic curves of the CHG index and TYG index for predicting new‐onset hypertension. The basic Model adjusted for sex, age, drinking, smoking, residence, BMI, education level, liver disease, chronic lung diseases, asthma, digestive disorders, dyslipidemia, kidney disease, arthritis, mental health disorders, memory‐related diseases, and cancer.

**TABLE 4:** Improvement in discrimination and risk reclassification for hypertension after adding the cholesterol, high‐density lipoprotein, and glucose index.

Model	C statistic estimate (95% CI)	*p*	NRI (continuous) estimate (95% CI)	*p*	IDI estimate (95% CI)	*p*
Basic model	0.624 (0.609–0.638)	Ref	Ref		Ref	
+DM	0.628 (0.613–0.642)	0.042	0.111 (0.073–0.149)	< 0.001	0.004 (0.002–0.005)	< 0.001
+TyG index	0.638 (0.623–0.652)	0.003	0.162 (0.110–0.214)	< 0.001	0.010 (0.007–0.013)	< 0.001
+CHG index	0.641 (0.626–0.655)	< 0.001	0.197 (0.145–0.249)	< 0.001	0.011 (0.009–0.014)	< 0.001

*Note:* The basic Model included sex, age, drinking status, smoking status, residence, BMI, educational level, liver disease, chronic lung disease, asthma, digestive disorders, dyslipidemia, kidney disease, arthritis, mental health disorders, memory‐related diseases, and cancer.

### 3.4 Subgroup and Sensitivity Analysis

Subgroup analyses were performed according to age, BMI, drinking status, and smoking status (Figure 3). Overall, the positive association between the CHG index and incident hypertension was generally consistent across the predefined subgroups. Although the association did not reach statistical significance among participants with obesity (BMI ≥ 28 kg/m^2^), the interaction test between BMI categories and the CHG index was not statistically significant (*P* for interaction > 0.05). Likewise, no significant interactions were observed for age, smoking status, or drinking status (all *P* for interaction > 0.05), indicating no reliable evidence that these factors modified the association between the CHG index and incident hypertension.

**FIGURE 3:**
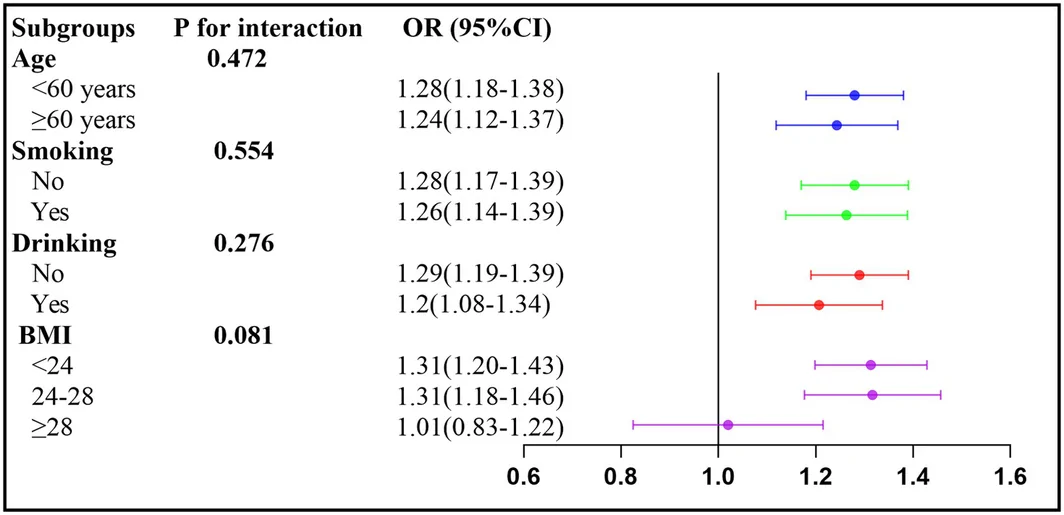
Subgroup analysis of the relationship between CHG index and new‐onset hypertension.

In sensitivity analyses, additional adjustment for laboratory variables (uric acid and C‐reactive protein) or for medication use (lipid‐lowering, glucose‐lowering, and antihypertensive agents) did not materially alter the results; the association between the CHG index and incident hypertension remained statistically significant in both models (Tables S4 and S5).

## 4 Discussion

To our knowledge, this large‐scale study is the first to evaluate the association between the CHG index and incident hypertension across different sexes and glycemic statuses. The results indicated a significant positive linear relationship between the CHG index and incident hypertension, with similar associations observed in both sexes. Although a significant association between the CHG index and incident hypertension was found across different glycemic statuses, no significant dose–response relationship was observed between the CHG index and new‐onset hypertension in the normal glycemic population. In addition, the CHG index improved the discrimination of the baseline model for incident hypertension and demonstrated predictive performance comparable to that of the TyG index. However, given the observational nature of this study, these findings should be interpreted with caution.

Hypertension and diabetes have a complex bidirectional relationship [17, 18]. The prevalence of hypertension is significantly higher in individuals with Type 2 diabetes, while the risk of developing Type 2 diabetes in individuals with hypertension is approximately 2.5 times that of those with normal blood pressure [19, 20, 21]. This close epidemiological association suggests that insulin resistance may play a key role in the pathogenesis of hypertension, providing an important theoretical basis for our research. Previous studies have confirmed that the TyG index is associated with hypertension risk, which is consistent with our findings [10, 22, 23, 24]. However, the novelty of our study lies in the fact that it is the first to reveal a significant correlation between the CHG index and incident hypertension and systematically compare the predictive efficacy of the CHG index and the TyG index.

Our findings suggest that the CHG index may serve as a useful marker of metabolic status associated with incident hypertension across different population subgroups. Although the CHG index demonstrated a predictive performance comparable to that of the TyG index, the logistic regression analyses showed a relatively stronger association between the CHG index and incident hypertension. This observation may be attributable to the incorporation of additional metabolic parameters, particularly high‐density lipoprotein cholesterol and total cholesterol, which may better capture metabolic abnormalities associated with hypertension development [25, 26]. Consistent with previous studies, our findings suggest that composite indices derived from the TyG index may provide complementary information beyond the TyG index alone. However, given the observational nature of this study, these findings should be interpreted as hypothesis‐generating. Further validation in independent external cohorts and prospective studies is warranted before the CHG index can be considered for risk prediction, risk stratification, or other clinical applications.

There is a significant correlation between the CHG index and new‐onset hypertension, with several potential pathophysiological mechanisms explaining this relationship. First, insulin resistance and hyperglycemia can lead to excessive activation of the renin‐angiotensin‐aldosterone system (RAAS), and the aberrant activation of RAAS can interfere with insulin signalling, creating a vicious cycle that ultimately triggers hypertension [17, 27]. Second, aldosterone and angiotensin II can upregulate serum and glucocorticoid‐regulated kinase 1 (SGK‐1), which activates the epithelial sodium channel, inhibiting the activity of endothelial nitric oxide synthase (eNOS) and reducing nitric oxide (NO) production, thereby impairing endothelial function and vascular relaxation [28, 29]. Lastly, hyperinsulinemia associated with metabolic insulin resistance can increase endothelin‐1 (ET‐1) synthesis through a mitogen‐activated protein kinase (MAPK)‐dependent signalling pathway, promoting vasoconstriction, arterial stiffness, and ultimately contributing to the development of hypertension [17, 30].

We must acknowledge that there are several limitations to this study. First, because this study is observational, we cannot establish causality, and observational studies themselves may have potential selection bias caused by participant recruitment and loss to follow‐up. Second, the hypertension diagnosis is based only on participants' self‐reports of physician diagnosis, which may lead to recall bias. Nevertheless, self‐reported disease endpoints are commonly applied in large cohort investigations. Validation evidence from the English Longitudinal Study of Ageing (ELSA), where self‐reports could be cross‐checked against medical records, demonstrated 77.5% concordance for self‐reported cardiovascular outcomes, suggesting acceptable reliability for population‐level analyses [31]. Still, misclassification may attenuate the observed association between CHG index and new‐onset hypertension in our study. Third, statistically, we applied multivariate logistic regression to calculate odds ratios (ORs) for outcome association assessment. Given that the cumulative incidence of new‐onset hypertension reached approximately 33% during the follow‐up period, the OR values tend to overestimate the true relative risk inherently. If precise time points of hypertension onset can be obtained in future research, modified Poisson regression with robust variance can be adopted to calculate risk ratios (RRs) for more accurate quantification of effect magnitudes. Fourth, despite adjusting for multiple covariates in our model, we cannot completely rule out residual confounding by factors related to diet, lifestyle, and daily activities. In particular, data on dietary salt intake and urinary sodium/potassium excretion, which are known risk factors for hypertension, were not included in our regression adjustments, and their absence may have influenced the observed association between the CHG index and new‐onset hypertension. Fifth, there was a certain degree of sample attrition in this study. A major source of exclusion was missing blood‐based biomarker data, as valid laboratory measurements were required to construct the CHG index. Although we compared baseline characteristics between included and excluded participants in Table S1, biomarker‐related missingness may not be entirely random. Participants who declined blood collection might differ in metabolic profiles and lifestyle behaviours from those who provided blood samples. Such non‐random missingness could introduce potential selection bias, which may partly distort effect estimates and restrict the generalizability of our findings. Finally, this study is only for the Chinese population aged 45 and older, and the results may not be generalizable to other populations. Therefore, further studies in a broader population sample and diverse demographic context are needed to verify our results in the future.

## 5 Conclusion

An elevated CHG index is significantly associated with new‐onset hypertension, particularly among males or individuals with abnormal glucose metabolism. After incorporating the CHG index into the baseline predictive model, the model's ability to distinguish new‐onset hypertension improved significantly, with predictive performance comparable to that of the TyG index.

## Author Contributions

**Huabin He:** investigation, writing – original draft, methodology, validation, visualization, writing – review and editing, software, data curation. **Jianhai Chen:** funding acquisition, writing – original draft, writing – review and editing, formal analysis, data curation.

## Funding

The authors have nothing to report.

## Ethics Statement

The study protocol was approved by the Ethics Review Committee of Peking University (IRB00001052‐11015), and all participants provided written informed consent.

## Consent

All authors agree to take full responsibility for the completeness and accuracy of the manuscript and have reviewed and approved the final published version.

## Conflicts of Interest

The authors declare no conflicts interests.

## Supporting information


**Figure S1:** Flow chart of the study population.
**Figure S2:** Calibration curve of Model 3 for predicting new‐onset hypertension, alongside Hosmer‐Lemeshow goodness‐of‐fit test (*χ* ^2^ = 13.146, df = 8, *p* = 0.107).
**Table S1:** Variance inflation factor (VIF) for covariates included in Model 3.
**Table S2:** Characteristics of CHARLS participants aged ≥ 45 years included versus not included in this study.
**Table S3:** Association between the TyG index and new‐onset hypertension.
**Table S4:** Association between CHG index and risk of new onset hypertension after further adjustment for UA and CRP based on Model 3.
**Table S5:** Association between CHG index and risk of new onset hypertension after further adjustment for medication use (lipid‐lowering, glucose‐lowering, and antihypertensive drugs) based on Model 3.

## Data Availability

The CHARLS data for our study can be publicly available at https://charls.pku.edu.cn/.

## References

[1] NCD Risk Factor Collaboration (NCD‐RisC) , “Worldwide Trends in Hypertension Prevalence and Progress in Treatment and Control From 1990 to 2019: A Pooled Analysis of 1201 Population‐Representative Studies With 104 Million Participants,” Lancet 398, no. 10304 (2021): 957–980, 10.1016/S0140-6736(21)01330-1. PMC8446938 34450083

[2] National Center for Cardiovascular Diseases & The Writing Committee of the Report on Cardiovascular Health and Diseases in China , “Summary of the China Cardiovascular Health and Disease Report 2023,” Chinese Journal of Circulation 39, no. 7 (2024): 625–660. 10.3967/bes2024.162 39401992

[3] M. Burnier and A. Damianaki , “Hypertension as Cardiovascular Risk Factor in Chronic Kidney Disease,” Circulation Research 132, no. 8 (2023): 1050–1063, 10.1161/CIRCRESAHA.122.321762. 37053276

[4] G. Gartlehner , E. B. Vander Schaaf , C. Orr , S. M. Kennedy , R. Clark , and M. Viswanathan , “Screening for Hypertension in Children and Adolescents: Updated Evidence Report and Systematic Review for the US Preventive Services Task Force,” JAMA 324, no. 18 (2020): 1884–1895, 10.1001/jama.2020.11119. 33170247

[5] B. M. Y. Cheung and C. Li , “Diabetes and Hypertension: Is There a Common Metabolic Pathway?,” Current Atherosclerosis Reports 14, no. 2 (2012): 160–166, 10.1007/s11883-012-0227-2. PMC3314178 22281657

[6] K.‐Z. Tsai , C.‐C. Chu , W.‐C. Huang , X. Sui , C. J. Lavie , and G.‐M. Lin , “Prediction of Various Insulin Resistance Indices for the Risk of Hypertension Among Military Young Adults: The CHIEF Cohort Study, 2014‐2020,” Cardiovascular Diabetology 23, no. 1 (2024): 141, 10.1186/s12933-024-02229-8. PMC11046748 38664804

[7] Z. Guo , X. Guo , H. Xu , et al., “Association Between Metabolic Score for Insulin Resistance (METS‐IR) and Hypertension: A Cross‐Sectional Study Based on NHANES 2007‐2018,” Lipids in Health and Disease 24, no. 1 (2025): 64, 10.1186/s12944-025-02492-y. PMC11843973 39985010

[8] D. Mo , P. Zhang , M. Zhang , H. Dai , and G. Wang , “Association Between the Atherogenic Index of Plasma and Incident Hypertension Across Different Blood Pressure States: A National Cohort Study,” Cardiovascular Diabetology 24, no. 1 (2025): 219, 10.1186/s12933-025-02775-9. PMC12093804 40399995

[9] Y. Zhang , P. Gesang , Y. Zhou , K. Ding , Y. Wan , and H. Xiong , “Relationship Between Lipid Levels, TyG, TyG‐BMI Index and Hypertension in Tibetan Population in Tibet, China Based on Restricted Cubic Spline Model,” BMC Public Health 24, no. 1 (2024): 3595, 10.1186/s12889-024-21160-x. PMC11673320 39731090

[10] M. Wang , T. Teng , N. Zhang , et al., “Derivatives of the Triglyceride‐Glucose Index and Their Association With Incident Hypertension in Prehypertensive Individuals: A 4‐Year Cohort Study Augmented by Mendelian Randomization,” Cardiovascular Diabetology 24, no. 1 (2025): 284, 10.1186/s12933-025-02813-6. PMC12247458 40646540

[11] A. Mansoori , M. Nosrati , M. Dorchin , et al., “A Novel Index for Diagnosis of Type 2 Diabetes Mellitus: Cholesterol, High Density Lipoprotein, and Glucose (CHG) Index,” J Diabetes Investig 16, no. 2 (2025): 309–314, 10.1111/jdi.14343. PMC11786182 39569998

[12] D. Mo , P. Zhang , M. Zhang , H. Dai , and J. Guan , “Cholesterol, High‐Density Lipoprotein, and Glucose Index Versus Triglyceride–Glucose Index in Predicting Cardiovascular Disease Risk: A Cohort Study,” Cardiovascular Diabetology 24 (2025): 116, 10.1186/s12933-025-02675-y. PMC11895360 40065297

[13] Y. Zhao , Y. Hu , J. P. Smith , J. Strauss , and G. Yang , “Cohort Profile: The China Health and Retirement Longitudinal Study (CHARLS),” International Journal of Epidemiology 43, no. 1 (2014): 61–68, 10.1093/ije/dys203. PMC3937970 23243115

[14] L. E. Simental‐Mendía , M. Rodríguez‐Morán , and F. Guerrero‐Romero , “The Product of Fasting Glucose and Triglycerides as Surrogate for Identifying Insulin Resistance in Apparently Healthy Subjects,” Metabolic Syndrome and Related Disorders 6, no. 4 (2008): 299–304, 10.1089/met.2008.0034. 19067533

[15] American Diabetes Association , “2. Classification and Diagnosis of Diabetes: Standards of Medical Care in Diabetes‐2021,” Diabetes Care 44, no. Suppl 1 (2021): S15–S33, 10.2337/dc21-S002. 33298413

[16] C. Liu , X. Liu , X. Ma , et al., “Predictive Worth of Estimated Glucose Disposal Rate: Evaluation in Patients With Non‐ST‐Segment Elevation Acute Coronary Syndrome and Non‐Diabetic Patients After Percutaneous Coronary Intervention,” Diabetology and Metabolic Syndrome 14, no. 1 (2022): 145, 10.1186/s13098-022-00915-9. PMC9535978 36203208

[17] G. Jia and J. R. Sowers , “Hypertension in Diabetes: An Update of Basic Mechanisms and Clinical Disease,” Hypertension 78, no. 5 (2021): 1197–1205, 10.1161/HYPERTENSIONAHA.121.17981. PMC8516748 34601960

[18] J. Wu , Q. Li , Y. Feng , et al., “Active Commuting and the Risk of Obesity, Hypertension and Diabetes: A Systematic Review and Meta‐Analysis of Observational Studies,” BMJ Global Health 6, no. 6 (2021): e005838, 10.1136/bmjgh-2021-005838. PMC8237743 34172487

[19] G. S. Wei , S. A. Coady , D. C. Goff, Jr. , et al., “Blood Pressure and the Risk of Developing Diabetes in African Americans and Whites: ARIC, CARDIA, and the Framingham Heart Study,” Diabetes Care 34, no. 4 (2011): 873–879, 10.2337/dc10-1786. PMC3064044 21346180

[20] Y. Wu , H. Hu , J. Cai , et al., “Association of Hypertension and Incident Diabetes in Chinese Adults: A Retrospective Cohort Study Using Propensity‐Score Matching,” BMC Endocrine Disorders 21, no. 1 (2021): 87, 10.1186/s12902-021-00747-0. PMC8082672 33926442

[21] J. Uddin , S. Zhu , G. Malla , et al., “Trends in Diagnosed Hypertension Prevalence by Geographic Region for Older Adults With and Without Diagnosed Diabetes, 2005–2017,” Journal of Diabetes and Its Complications 36, no. 7 (2022): 108208, 10.1016/j.jdiacomp.2022.108208. PMC10142383 35597703

[22] F. Xin , S. He , Y. Zhou , X. Jia , Y. Zhao , and H. Zhao , “The Triglyceride Glucose Index Trajectory Is Associated With Hypertension: A Retrospective Longitudinal Cohort Study,” Cardiovascular Diabetology 22, no. 1 (2023): 347, 10.1186/s12933-023-02087-w. PMC10725029 38102704

[23] F. Zhang , Y. Zhang , Z. Guo , et al., “The Association of Triglyceride and Glucose Index, and Triglyceride to High‐Density Lipoprotein Cholesterol Ratio With Prehypertension and Hypertension in Normoglycemic Subjects: A Large Cross‐Sectional Population Study,” Journal of Clinical Hypertension 23, no. 7 (2021): 1405–1412, 10.1111/jch.14305. PMC8678664 34118112

[24] Y. Pan , S. Zou , Y. Xu , et al., “Is There Any Association Between Early Trimester Triglyceride‐Glucose Index and Incidence of Hypertensive Disorder of Pregnancy and Adverse Pregnancy Outcomes?,” Front Endocrinol 14 (2023): 1093991, 10.3389/fendo.2023.1093991. PMC10025371 36950677

[25] J. Hong , R. Zhang , H. Tang , S. Wu , Y. Chen , and X. Tan , “Comparison of Triglyceride Glucose Index and Modified Triglyceride Glucose Indices in Predicting Cardiovascular Diseases Incidence Among Populations With Cardiovascular‐Kidney‐Metabolic Syndrome Stages 0‐3: A Nationwide Prospective Cohort Study,” Cardiovascular Diabetology 24, no. 1 (2025): 98, 10.1186/s12933-025-02662-3. PMC11871812 40022122

[26] Y. Qiao , Y. Wang , C. Chen , Y. Huang , and C. Zhao , “Association Between Triglyceride‐Glucose (TyG) Related Indices and Cardiovascular Diseases and Mortality Among Individuals With Metabolic Dysfunction‐Associated Steatotic Liver Disease: A Cohort Study of UK Biobank,” Cardiovascular Diabetology 24, no. 1 (2025): 12, 10.1186/s12933-024-02572-w. PMC11730479 39806394

[27] G. Jia , A. Whaley‐Connell , and J. R. Sowers , “Diabetic Cardiomyopathy: A Hyperglycaemia‐ and Insulin‐Resistance‐Induced Heart Disease,” Diabetologia 61, no. 1 (2018): 21–28, 10.1007/s00125-017-4390-4. PMC5720913 28776083

[28] A. C. Montezano and R. M. Touyz , “Reactive Oxygen Species, Vascular Noxs, and Hypertension: Focus on Translational and Clinical Research,” Antioxidants & Redox Signaling 20, no. 1 (2014): 164–182, 10.1089/ars.2013.5302. PMC3880913 23600794

[29] M. Yang , Q. Shangguan , G. Xie , G. Sheng , and J. Yang , “Oxidative Stress Mediates the Association Between Triglyceride‐Glucose Index and Risk of Cardiovascular and All‐Cause Mortality in Metabolic Syndrome: Evidence From a Prospective Cohort Study,” Front Endocrinol 15 (2024): 1452896, 10.3389/fendo.2024.1452896. PMC11368748 39229375

[30] Y.‐J. Lin , C. F. Kwok , C. C. Juan , et al., “Angiotensin II Enhances Endothelin‐1‐Induced Vasoconstriction Through Upregulating Endothelin Type A Receptor,” Biochemical and Biophysical Research Communications 451, no. 2 (2014): 263–269, 10.1016/j.bbrc.2014.07.119. 25088996

[31] C. Cui , L. Liu , T. Zhang , et al., “Triglyceride‐Glucose Index, Renal Function and Cardiovascular Disease: A National Cohort Study,” Cardiovascular Diabetology 22, no. 1 (2023): 325, 10.1186/s12933-023-02055-4. PMC10685637 38017519

