# Supplementary material for: Association Between Cholesterol‐HDL‐Glucose (CHG) Index and New‐Onset Hypertension Across Different Sex and Glycemic Status: A Longitudinal Study With 9‐Year Follow‐Up
Source: Endocrinol Diabetes Metab. 2026 Nov 3;9(6):e70365. doi: 10.1002/edm2.70365 (PMC13731164; doi:10.1002/edm2.70365)
Supplement: Supplementary file 1 [file EDM2-9-e70365-s001.docx]

# Association between cholesterol-HDL-glucose (CHG) index and new-onset hypertension across different sex and glycemic status: a longitudinal study with 9-year follow-up

## Supplementary Figures and tables

**Supplementary Figure 1:** Flow chart of the study population.

**Supplementary Figure 2:** Calibration curve of Model 3 for predicting new‑onset hypertension, alongside Hosmer‑Lemeshow goodness‑of‑fit test (χ²=13.146, df=8, P=0.107).

**Table S1.** Variance inflation factor (VIF) for covariates included in Model 3

**Table S2.** Characteristics of CHARLS participants aged ≥45 years included versus not included in this study

**Table S3.** Association between the TyG index and new-onset hypertension

**Table S4.** Association between CHG index and risk of new onset hypertension after further adjustment for UA and CRP based on model 3.

**Table S5.** Association between CHG index and risk of new onset hypertension after further adjustment for medication use (lipid‑lowering, glucose‑lowering, and antihypertensive drugs) based on model 3.

**Supplementary Figure 1:** Flow chart of the study population.

**Supplementary Figure 2:** Calibration curve of Model 3 for predicting new‑onset hypertension, alongside Hosmer‑Lemeshow goodness‑of‑fit test (χ²=13.146, df=8, P=0.107).

**Table S1.** Variance inflation factor (VIF) for covariates included in Model 3

| Variable | GVIF | Df | GVIF^(1/(2*Df)) |
| --- | --- | --- | --- |
| CHG_quartiles | 1.119119 | 3 | 1.018934 |
| Age | 1.443083 | 1 | 1.201284 |
| Sex | 2.370947 | 1 | 1.539788 |
| BMI | 1.205104 | 2 | 1.047746 |
| Drinking | 1.305709 | 1 | 1.142676 |
| Smoking | 1.262331 | 1 | 1.123535 |
| Education level | 1.350753 | 3 | 1.051387 |
| Residence | 1.172378 | 1 | 1.082764 |
| Dyslipidemia | 1.062326 | 1 | 1.030692 |
| DM | 1.006557 | 1 | 1.003273 |
| Digestive disorders | 1.151977 | 1 | 1.073302 |
| Chronic lung diseases | 1.023034 | 1 | 1.011452 |
| Liver disease | 1.072505 | 1 | 1.035618 |
| Cancer | 1.025246 | 1 | 1.012545 |
| Kidney disease | 1.083857 | 1 | 1.041084 |
| Memory-related diseases | 1.026519 | 1 | 1.013173 |
| Mental health disorders | 1.037649 | 1 | 1.01865 |
| Arthritis | 1.08587 | 1 | 1.042051 |
| Asthma | 1.129769 | 1 | 1.062906 |
| Systolic blood pressure | 2.163444 | 1 | 1.470865 |
| Diastolic blood pressure. | 2.127152 | 1 | 1.458476 |

**Table S2.** Characteristics of CHARLS participants aged ≥45 years included versus not included in this study

| Participants | Included | Excluded |
| --- | --- | --- |
| Participants, N | 6555 | 10717 |
| Age, years | 57.30 ± 8.46 | 60.94 ± 9.65 |
| Male, n (%) | 3016 (46.0) | 5176 (48.3） |
| Smoking, n (%) | 2517 (38.4) | 4211 (39.3） |
| Drinking, n (%) | 2002 (30.6) | 3858 (36.1） |
| Marital status (n, %) | | |
| Partnered | 5953 (90.8) | 9500 (88.7) |
| Single | 602 (9.2) | 1211 (11.3) |
| Rural (vs urban), n (%) | 5569 (85.0) | 8766 (81.8) |
| Educational level, n (%) | | |
| Illiterate | 2971 (45.3) | 4479 (41.8) |
| Primary school | 1445 (22.1) | 2840 (26.5) |
| Middle school | 1432 (21.9) | 2507 (23.4) |
| High school or above | 702 (10.7) | 889 (8.3) |
| BMI, (kg/m2) | 23.0 (21.0, 25.0) | 23.3 (19.7, 26.3) |

**Table S3.** Association between the TyG index and new-onset hypertension

| Categories | Mode 1 | | Mode 2 | | Mode 3 | |
| --- | --- | --- | --- | --- | --- | --- |
|  | **OR (95% CI)** | **P value** | **OR (95% CI)** | **P value** | **OR (95% CI)** | **P value** |
| Quartiles |  |  |  |  |  |  |
| Q1 | Ref |  | Ref |  | Ref |  |
| Q2 | 1.19(1.02-1.39) | 0.026 | 1.17(1.00-1.37) | 0.055 | 1.15(0.99-1.35) | 0.075 |
| Q3 | 1.49(1.28-1.74) | <0.001 | 1.38(1.18-1.61) | <0.001 | 1.35(1.16-1.58) | <0.001 |
| Q4 | 1.92(1.65-2.23) | <0.001 | 1.73(1.48-2.02) | <0.001 | 1.60(1.36-1.89) | <0.001 |

**Table S4.** Association between CHG index and risk of new onset hypertension after further adjustment for UA and CRP based on model 3.

| Categories | Total | | Male | | Female | |
| --- | --- | --- | --- | --- | --- | --- |
|  | **aOR (95% CI)** | **P value** | **aOR (95% CI)** | **P value** | **aOR (95% CI)** | **P value** |
| Continues (Per SD increase) | 1.25(1.17-1.33) | <0.001 | 1.28(1.17-1.40) | <0.001 | 1.21(1.11-1.33) | <0.001 |
| Quartiles |  |  |  |  |  |  |
| Q1 | Ref |  | Ref |  | Ref |  |
| Q2 | 1.33(1.14-1.57) | <0.001 | 1.1(0.87-1.40) | 0.433 | 1.55(1.25-1.94) | <0.001 |
| Q3 | 1.59(1.35-1.86) | <0.001 | 1.54(1.22-1.94) | <0.001 | 1.62(1.29-2.03) | <0.001 |
| Q4 | 1.81(1.52-2.15) | <0.001 | 1.91(1.49-2.46) | <0.001 | 1.7(1.34-2.17) | <0.001 |

**Table S5.** Association between CHG index and risk of new onset hypertension after further adjustment for medication use (lipid‑lowering, glucose‑lowering, and antihypertensive drugs) based on model 3.

| Categories | Total | | Male | | Female | |
| --- | --- | --- | --- | --- | --- | --- |
|  | **aOR (95% CI)** | **P value** | **aOR (95% CI)** | **P value** | **aOR (95% CI)** | **P value** |
| Continues (Per SD increase) | 1.15(1.10- 1.23) | <0.001 | 1.21(1.09- 1.33) | <0.001 | 1.12(1.02- 1.22) | 0.017 |
| Quartiles |  |  |  |  |  |  |
| Q1 | Ref |  | Ref |  | Ref |  |
| Q2 | 1.27(1.06- 1.53) | 0.010 | 1.10(0.83- 1.45) | 0.517 | 1.46(1.13- 1.88) | 0.003 |
| Q3 | 1.31(1.08- 1.57) | 0.005 | 1.21(0.92- 1.60) | 0.165 | 1.42(1.10- 1.84) | 0.008 |
| Q4 | 1.49(1.24- 1.80) | <0.001 | 1.66(1.26- 2.20) | <0.001 | 1.4(1.08- 1.82) | 0.011 |
